# Welding Fume Exposure and Epigenetic Alterations: A Systematic Review

**DOI:** 10.3390/ijerph16101745

**Published:** 2019-05-17

**Authors:** Veruscka Leso, Ilaria Vetrani, Ilaria Della Volpe, Caterina Nocera, Ivo Iavicoli

**Affiliations:** Section of Occupational Medicine, Department of Public Health, University of Naples Federico II, Via S. Pansini 5, 80131 Naples, Italy; ilariavetrani20@gmail.com (I.V.); ilaria.della@virgilio.it (I.D.V.); caterina.nocera88@gmail.com (C.N.); ivo.iavicoli@unina.it (I.I.)

**Keywords:** welding fumes, occupational exposure, particulate matter, epigenetic effects, epigenome, DNA methylation, health effects, biological indicators, risk assessment, risk management

## Abstract

Epigenetics are heritable changes in gene expression not coded in the DNA sequence, which stand at the interface between the genome, environmental exposure and development. From an occupational health perspective, epigenetic variants may link workplace exposures and health effects. Therefore, this review aimed to overview possible epigenetic effects induced by welding fumes on exposed workers and health implications. A systematic search was performed on Pubmed, Scopus, and ISI Web of Knowledge databases. DNA methylation changes have been reported in genes responsible for the cardiac autonomic function and coagulation, i.e., LINE-1, GPR133 and F2RL3, in mitochondrial-DNA-sequences involved in the regulation of energy-generation/redox-signaling, as well as in inflammatory activated genes, i.e., iNOS. However, the limited number of retrieved articles, their cross-sectional nature, the lack of a suitable qualitative-quantitative exposure assessment, and the heterogeneity of biological-outcomes investigated, prevent the extrapolation of a definite causal relationship between welding fumes and epigenetic phenomena. Future studies should clarify the function of such epigenetic alterations as possible markers of occupational exposure and early effect, dose-response relationships, and underlying molecular mechanisms. Overall, this may be helpful to guide suitable risk assessment and management strategies to protect the health of workers exposed to welding fumes.

## 1. Introduction

The term “epigenetics” defines all the heritable changes in gene expression not coded in the DNA sequence itself, which are able to control development, tissue differentiation and cellular responsiveness [1]. DNA methylation, post-translational histone modifications, and chromatin remodeling are the most used mechanisms that are able to initiate and sustain epigenetic information. Overall, this is controlled by the genome sequence, environmental exposure, and stochasticity [2]. As such, epigenetics stands at the interface of the genome, environmental exposure and development.

In this regard, epigenetic imbalances were reported to be induced by the exposure to environmental pollutants, including particulate matter (PM), combustion byproducts, contaminating trace metals and residual organic compounds [3,4,5,6,7,8]. In particular, epigenetic effects have been triggered by environmental metals, arsenic in particular, but also iron, lead, nickel, cadmium, chromium and manganese, following in the utero, general living and occupational exposures [3,9,10,11,12,13,14,15,16,17,18,19]. Such alterations attracted great scientific interests as metal exposure is known to have important health impacts, and, in many cases, the mechanisms through which these health effects occur at the cellular and molecular level are poorly understood [3]. In this scenario, epigenetic phenomena may elucidate the link between risk factors and biological alterations/disease manifestation therefore characterizing a promising biological indicator of early effect and disease susceptibility.

From an occupational health perspective, such findings pose some questions on possible epigenetic effects induced by the exposure to welding fumes. A metal-rich mixture of fine and ultrafine PM, in fact, is generated by the extreme heat produced by welding, whose composition depends on the characteristics of the base metal as well as on the type of welding techniques applied [20]. Epidemiological studies have demonstrated that chronic exposure to welding fumes is associated with respiratory health effects, such as asthma, bronchitis, lung function changes, and cardiovascular diseases [20,21]. Welding fumes have also been classified as carcinogenic to humans (Group 1) by the International Agency for Research on Cancer (IARC) [22]. Unfortunately, mechanisms linking welding fume exposure to adverse health outcomes are still not fully understood. In this regard, it cannot be excluded that welding fumes may induce epigenetic alterations that may affect the gene expression profile, therefore characterizing a potential linking mode of action.

The aim of our review was to provide a comprehensive overview on possible epigenetic effects induced by welding fumes in occupationally exposed subjects and their possible implications for workers’ health. This was finalized to gain insight into possible molecular mechanisms underlying health effects induced by chronic exposures to welding fumes in workplaces and to define early biological alterations that may function as possible biomarkers of effect or susceptibility in exposed welders. Overall, this review may reveal a useful means to unlock the relationship between workers’ genome, occupational exposure and disease risk and may be helpful to point out biological mechanisms and predictors of disease that may guide the adoption of suitable preventive measures to protect the health of exposed workers.

## 2. Materials and Methods

**Figure 1:**
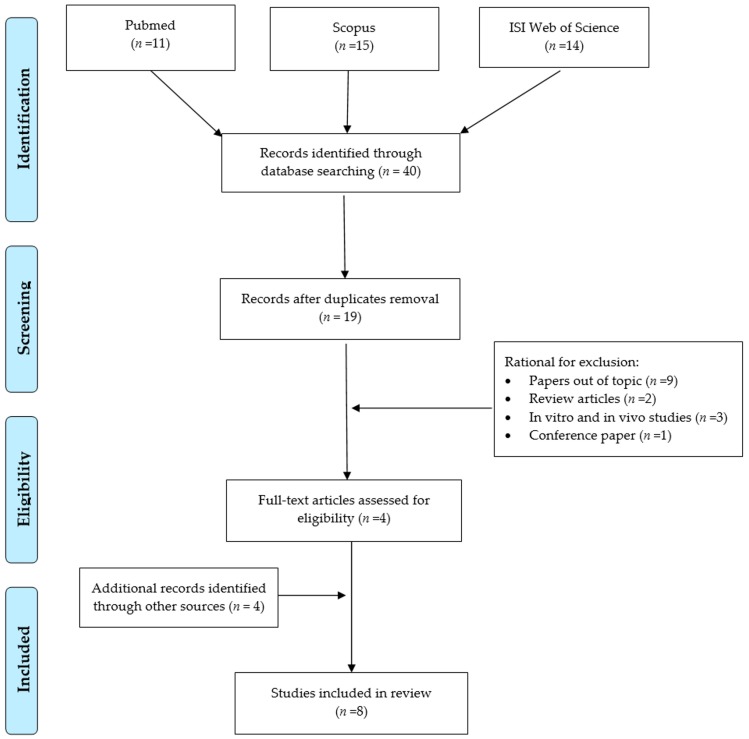
Flow diagram of literature search. Search string employed in the three different databases: Pubmed: ((((“Welding Fumes”) OR welder OR welding))) AND epigen*; Scopus: (TITLE-ABS-KEY (“Welding Fumes”) OR TITLE-ABS-KEY (welder) OR TITLE-ABS-KEY (welding)) AND (TITLE-ABS-KEY (epigen*)); ISI Web of Science: ALL = (“Welding Fumes” OR welder OR welding) AND ALL = (epigen*).

A systematic literature search was performed according to the Preferred Reporting Items for Systematic Reviews and Meta-Analyses Statement (PRISMA) criteria [23]. PubMed, Scopus and ISI Web of Science databases were searched to identify studies addressing epigenetic effects induced by occupational welding fume exposure and published until 6 April 2019 (Figure 1). A preliminary search was carried out for the terms “welding fumes or welder or welding” to assess the exposure context and “epigen*” as the outcome of the investigation, which were combined with the Boolean operator “AND”. Three of the authors independently examined all titles and abstracts retrieved and selected articles that met the inclusion criteria. These included all types of human peer-reviewed research articles, (i.e., cross-sectional, cohort, case-control studies), published in English and exploring the welding fume exposure—epigenetic alterations relationship in occupational contexts. Articles published in languages other than English, in vitro and in vivo experimental studies, review and conference papers, as well as publications not focusing on the association between epigenetic effects induced by welding fume exposure in workers were excluded as not suitable for the review. The preliminary search retrieved 11, 15 and 14 references through PubMed, Scopus and ISI Web of Science databases, respectively, for a total of 40 articles. After removal of the duplicates, 19 articles remained. Among those, studies that did not meet the inclusion criteria were excluded according to the following reasons: nine out of 19 were removed because studies out of the topic from the title and abstract analysis; two out of 19 were excluded as review articles, one out of 19 as a conference paper; and three out of 19 as experimental studies on in vitro or in vivo models. Indeed, four publications could be identified in this preliminary phase. Therefore, we extended our search including the following terms “welding fumes or welder or welding”, individually combined with “DNA methylation”, “histone acetylation”, and “non coding RNA”, that allowed including two additional papers. The assessment of the reference list accompanying published articles was employed to supplement the pool of relevant publications, and allowed the inclusion of two further eligible articles. All full texts of the articles considered valuable for the aim of our review were obtained and a critical evaluation was performed. Overall, our search retrieved a total of eight publications for review.

## 3. Results

The following paragraphs will attempt to summarize epigenetic effects induced by welding fume exposure in different occupational settings. All the reviewed studies focused on DNA methylation changes, a covalent modification of the nucleotide cytosine at the 5′ position, recognized as alterations of paramount importance as generally associated with gene silencing. Possible implications that such effects may have on biological alterations related to cardiovascular or inflammatory effects have been addressed (Table 1).

### 3.1. Epigenetic Changes and Cardiovascular Outcomes

Several air pollution studies have suggested that both acute and cumulative exposures to fine PM (PM_2.5_) may affect cardiac autonomic function, through the alteration of sympathetic and parasympathetic output [24,25,26,27]. In this regard, heart rate variability (HRV), which measures the variation in the time interval between heartbeats, is a noninvasive and sensitive indicator of cardiac autonomic function and has been frequently used to assess the cardiac effects of environmental exposures as a prognostic marker of various forms of cardiovascular diseases (CVDs).

Epigenetic regulation, such as DNA methylation, may play an important role in the alterations of cardiac autonomic responses, therefore representing a possible link between environmental exposure and heart effects (Table 1). In this regard, to assess the cardiac alterations and epigenetic changes in response to metallic welding fumes, Fan et al. [28] investigated the short-term effects of five h-welding PM_2.5_ exposure in a cohort of occupational US welders on the methylation status of short and long interspersed nucleotide genome elements, Alu and LINE-1, respectively, as well as the possible mediation role of such changes in affecting HRV. Alu and LINE-1 elements are widely represented across the human genome. Several studies have shown that decreased methylation in LINE-1 elements is associated with ischemic heart disease and stroke, as well as with CVD risk factors, suggesting a potential mediating function of DNA methylation in CVDs [29,30,31,32]. The authors found a significant positive relationship between PM_2.5_ exposure and LINE-1 methylation levels, while no significant association was determined on Alu. Such epigenetic changes could be related to the PM_2.5_ induced oxidative stress, that may affect the DNA methyltransferase function to reduce the methylation process on the DNA molecule [33]. An acute decline of HRV was also reported, although the authors failed to demonstrate a significant association between such effect and LINE-1 methylation. However, it should be noticed that such relation was tested after few hours of welding fume exposure, therefore limiting the power of the study in defining the link between LINE-1 methylation levels and adverse cardiac outcomes. These results only partially confirmed those reported by Kile et al. [34] that found no association between welding fume PM_2.5_ and Alu or LINE-1 methylation, with respect to both acute and chronic conditions of exposure in US boilermakers involved in cutting and welding metal plates [34].

More recently, Zhang et al. [35] investigated the epigenetic variants induced by PM_2.5_ welding exposure associated with cardiac autonomic changes measured with two innovative markers, the acceleration and deceleration capacity. A significant negative association between methylation at one CpG located in the body of the GPR133 gene on chromosome 12 and the heart rate deceleration capacity could be determined. These findings suggest a potential role of the GPR133 gene in the cardiac autonomic dysfunction through possible effects on the heart rate deceleration capacity, which has been previously identified as a predictor of cardiovascular mortality among patients affected by myocardial infarction [36,37,38]. Methylation in the gene body is often positively associated with GPR133 gene expression [39]. This may induce the activation of the adenylate cyclase and the G protein cascade pathways in the heart cells, therefore augmenting the intracellular cAMP production that may, in turn, influence heart rate modulation. However, further research is needed to define the potential role of detected methylation changes in inducing cardiac autonomic dysfunctions in welders. 

The possible mechanistic pathway underlying the association between welding fume exposure and CVDs through possible epigenetic effects on the coagulation factor II (thrombin) receptor-like 3 gene (F2RL3) expression was studied by Hossain et al. [40]. F2RL3 is expressed on chromosome 9 in different cell types, including circulating leucocytes [41]. It encodes for a cell surface protein, the thrombin protease-activated receptor 4 (PAR-4), involved in the immune function, blood coagulation as well as in the regulation of vascular endothelial cell activity [41,42,43]. Exposure to welding fumes was reported to be associated with hypomethylation of F2RL3, at the CpG_2, CpG_4 and CpG_5 sites. These epigenetic changes may be related to an increased expression of this gene that could result in enhanced inflammation and coagulation, thus representing a possible CVD biomarker. The effects on F2RL3 methylation were found at low-to-moderate levels of welding fume exposure and suggest that welders might be at risk for CVDs, despite precautions taken to control reduce exposure levels. The number of years working as welders was associated with the hypomethylation of CpG_4, although this association became non-significant in models adjusted for confounding factors, i.e., smoking and age. Additionally, the authors demonstrated that hypomethylation of CpG_4 had the strongest association with exposure to respirable dust, indicating that this particular site is a main target for the epigenetic welding fume effects.

In the cardiovascular system, mitochondria play a key role for the regulation of energy generation and redox signaling of cells. They are the primary targets of oxidative stress in response to exogenous exposures, and mitochondrial DNA (mtDNA) is particularly vulnerable to such insult [44]. Recently, DNA methylation machinery was found in mitochondria [45,46] suggesting that the epigenetic phenomena may control functions and biogenesis of these organelles [47]. However, there is still limited knowledge on how environmental exposures, including occupational contacts, can modify the mtDNA epigenetics and consequent implication for CVDs. Byun et al. [48] examined methylation changes in the blood mtDNA of US boilermaker exposed to PM_2.5_ in their welding activities. Two regions of the mitochondrial genome were explored. These included the D-loop promoter, involved in the initiation of the transcription process for all the mitochondrial genome, and the mitochondrially encoded tRNA phenylalanine (MT-TF) and mitochondrially encoded 12S ribosomal RNA (MT-RNR1) genes coding for a tRNA and a 12S ribosomal RNA essential for protein synthesis. The authors also investigated the relationship between such changes and HRV as a CVD biomarker. The mean DNA methylation level of the D-loop region was significantly lower in workers who were highly exposed to PM. The mean level of DNA methylation within the D-loop region, in fact, was 2.36% and 2.22% at PM_2.5_ concentrations of 0.15 and 0.38 mg/m^3^ in pre- and post- welding work, respectively. No significant associations could be observed between the PM exposure and MT-TF and MT-RNR1 methylation. Regarding possible implications of such changes on HRV, participants with higher mtDNA methylation levels, were more susceptible to the effect of PM_2.5_ on heart rate variability measures, although a direct effect of blood mtDNA methylation on HRV could not be demonstrated.

More recently, Xu et al. [49] investigated the effects of occupational exposure to particle-containing welding fumes on different biomarkers of the mtDNA function and the role of such alterations in affecting the cardiovascular response, measured as blood pressure changes. An impact on mtDNA was demonstrated as welders had higher mtDNA copy numbers and lower DNA methylation of the D-loop and MT-TF compared to controls. The authors failed to demonstrate a dose-response relationship between personal respirable dust concentrations and D-loop or MT-TF methylation, and no association between mtDNA methylation and years of working as welders. To assess the interaction between the relative number of unmethylated D-loop and MT-TF regions on systolic and diastolic blood pressure, enrolled welders were stratified into low and high mtDNA function groups according to the median of relative number of unmethylated D-loop and MT-TF regions. Interestingly, significantly higher systolic blood pressure could be detected in both groups with low mtDNA function compared to controls, but not in the high function group with a greater relative number of active (unmethylated) D-loop and MT-FT regions.

### 3.2. Epigenetic Effects and Inflammatory Responses

Kile et al. [34] assessed the acute and chronic metal-rich PM_2.5_ exposure on DNA methylation changes in the inducible nitric oxide synthase gene (iNOS) which is involved in the production of nitric oxide and plays an important role in a variety of cardio-pulmonary processes [50,51] (Table 1). The authors found that particulate exposure was associated with a methylation increase of 0.25% within the promoter region of iNOS gene for every 1 mg/m^3^ enhancement in PM_2.5_. Particularly, when comparing data from individuals who were welding during the day of collection, with those obtained from employees who were present at the union hall, but did not participate in welding activities, a stronger response was evident in the first group with respect to those workers exposed to the background PM_2.5_. Additionally, the PM_2.5_–iNOS methylation association was greater when workers who wore respirators were excluded from the investigated cohort. A small increase in iNOS methylation was also determined according to chronic exposures assessed as the number of years worked as boilermakers. However, it remains to be elucidated whether the changes in DNA methylation are true epigenetic effects or a consequence of the systemic inflammation derived from inhaling fine PM [34].

Li et al. [52] investigated the relationship between low-to-moderate occupational exposure to particles from welding fumes and cancer-related biomarkers, i.e., oxidative stress DNA adducts, changes in telomere length, and alterations in DNA methylation. While no differences between exposed subjects and controls were detected for the first two parameters, welders showed higher methylation of the tumor suppression adenomatous polyposis coli (APC) gene, which regulates the signaling pathway responsible for the control of cell growth [53]. Lung cancer patients, as well as patients with other types of epithelial cancers, showed hypermethylation of the APC promoter compared to healthy controls [54]. The mechanism of particle exposure-induced DNA methylation was not clearly understood, but it has been suggested that several pathways could be involved. Oxidative stress may be one possible mechanism for PM-induced hypermethylation, but also the disturbance of the one carbon metabolism that produces methyl groups for DNA methylation cannot be ruled out [55]. The authors found no association between genotoxicity, or epigenotoxicity, and respirable PM concentrations, suggesting that the epigenetic effects measured could better reflect long-term alterations.

Searles Nielsen et al. [56] investigated whether DNA methylation may be involved in the causal pathway between welding fume exposure and parkinsonism. The authors could demonstrate that parkinsonism cases, defined as those subjects having the highest scores at the Unified Parkinson Disease Rating Scale (UPDRS3), showed a significant inverse association between methylation in the gene coding for iNOS in humans, NOS2, and parkinsonism prevalence compared to controls, specifically at CpG site 8329 located in an exonic splicing enhancer of NOS2. These associations were not significant for the intermediate UPDRS3 group. The analysis of the NOS2 CpG site 8329 methylation in relation to welding fume exposure showed an inverse association between the total duration of exposure and such epigenetic effects in workers with less than 10 years of cumulative exposure, with a 0.16% drop in methylation per year of exposure. Lower methylation of the gene coding for iNOS was associated with greater signs of parkinsonism among workers from welding worksites, suggesting that inflammation mediated by iNOS may possibly contribute to the high prevalence of parkinsonism observed previously in workers exposed to welding fume. However, longitudinal studies are necessary to verify such preliminary results.

## 4. Discussion

This review represents the first attempt to provide an overview on possible early epigenetic changes induced by welding fume exposure and their possible link with biological alterations, disease development and progression. Although reviewed articles demonstrated possible DNA methylation changes in subjects occupationally exposed to welding fumes, the limited number of retrieved studies, currently, do not allow extrapolating definite conclusions on the function of such alterations as possible early biomarkers of cardiovascular or inflammatory effects.

This may be partly due to some biases intrinsically related to the experimental design of the studies. In fact, all the investigations were cross-sectional in nature, and this aspect prevents conclusions with regard to the causal-relationship between occupational exposure, epigenetic effects and biological phenotypes. Moreover, the limited number of enrolled subjects, the poor information concerning the composition of the PM exposure in welding activities, as well as the not always appropriate length of investigation, i.e., acute compared to chronic exposures [28,48] may preclude adequate extrapolation. The heterogeneity of epigenetic effects analyzed, and the diverse biological associated outcomes, i.e., HRV [28,30,35], blood pressure [49], inflammatory status [30,40], parkinsonism [56], undoubtedly, makes it difficult to compare the results, thus preventing firm conclusions.

Additionally, all the reviewed articles were focused exclusively on one epigenetic mechanism, DNA methylation. This is because of the stable nature of DNA methylation, and the availability of several technologies to determine the presence of methylated cytosines [3]. However, such kind of alterations may not be sufficiently representative of the epigenome phenomena induced by welding fume exposure. Unlike genetic studies, where the genotype can be consistently determined from any available diploid cell, the epigenome is tissue, cell and context specific, and so these considerations must be taken into account to understand exposure impacts on epigenetics [3]. In this regard, the employment of mixed blood cell-type population in the DNA methylation analyses may characterize another investigation bias [34,40,48,52]. Methylation patterns, in fact, may be limited to only specific cells within a sample, and the effects may be masked when more cellular types are included. Moreover, peripheral blood may only be considered a proxy for other tissues target of welding fume actions, i.e., the lungs, as well as the cardiovascular-system. In this sense, advances in biostatistical methodology should be pursued to support the control of such a number of confounding factors. Models able to control also for the inherent genetic variability (which may influence epigenetic programming and effects), intrapersonal variation over time, as well as possible additive effects of co-exposures that may occur in general living or occupational settings or derived from lifestyle habits, i.e., the ozone production in gas phase during the welding process, or smoking habit, should be developed [49,57,58,59].

Furthermore, although significant differences in epigenetic changes emerged from exposed workers compared to controls [40], no definite dose-response relationships could be defined from available data. A possible dose-response association in PM_2.5_ exposure was suggested by Kile et al. [34] who found a marginally greater DNA methylation response to acute welding fume exposure in workers directly performing welding activities, compared to those just exposed to the PM_2.5_ background concentrations in the practice hall, but not directly exposed to fumes. Comparably, the relation between methylation levels and chronic exposure, assessed as years worked as welders, could not be definitively extrapolated [34,40,52]. Therefore, ulterior studies seem necessary to define such dose-response relationships with the aim also to identify adequate limit values for occupational exposure. Additionally, in workplace settings, welding activities may characterize a relevant source of fine and ultrafine particles [49] whose toxicity may be related, among other factors, also to their aerodynamic diameter. Lower sized particles, in fact, may have a different toxicological profile compared to particles with the same chemical composition, but a greater aerodynamic diameter. Future studies should provide data on the size of particles produced by different welding technologies and the relationship with specific epigenetic effects. This aspect may be important to be taken into consideration when interpreting effect data. Moreover, possible association between PM qualitative metal-based composition, in terms of co-exposure to a mixture of diverse metals, and epigenetic effects should be elucidated. These different exposure features may be responsible for inconsistent results obtained in different studies addressing methylation in the same loci [28,34]. Therefore, quantitative and qualitative characterization of the exposures, including the analysis of particle size and chemical composition of the PM, seems absolutely important to understand the causative relationship between occupational exposure and epigenetic effects. Moreover, the personal protective equipment function in reducing levels of exposure and consequently the amount of epigenetic variants should be deeply investigated, as only one study in our review adjusted epigenetic findings for respirator use during sampling, showing also a stronger association between PM exposure and methylation levels when workers who wore respirators were excluded from the analysis [34].

Some open questions remain concerning de novo and maintenance methylation mechanisms through which environmental exposures trigger epigenetic alterations. Nonetheless, several mechanisms for environmental exposure-related epigenetic alterations should be considered. A number of reports have suggested that oxidative stress may determine epigenetic profile alterations [30,60]. In fact, the ability of the methyltransferase to decrease DNA methylation may be affected by PM-induced oxidative stress [33]. On the other side, the detection of demethylated mtDNA D-loop in response to welding fume exposure was associated with an increased expression of an oxidative phosphorylation enzyme, which may positively regulate the generation of ATP [50]. This may be interpreted as a mtDNA compensatory response to the oxidative stress induced by welding fumes, through the enhancement of energy production aimed to maintain normal cellular function [61,62,63]. Future studies should be focused at elucidating whether methylation changes may be the direct effect of welding fumes or mediated by the inflammatory action exerted by such exposure [34].

Ongoing efforts should continue to gain insight into the modes through which the functional effects of epigenetic variants operate, in terms of genetic, molecular and biochemical processes, in order to better understand the possible role of such effects as mediators in possible adverse health outcomes. In this regard, gene expression changes related to epigenetic alterations should be addressed. Particular attention should be focused on understanding the epigenome wide association with possible respiratory, cardiovascular, as well as carcinogenic disease pathways induced by welding fume exposure and the specificity of epigenetic effects for peculiar patterns of disease. Additionally, comparing results from different kinds of metal rich occupational PM exposure, i.e., during welding activities, in foundry tasks, as well as due to vehicular traffic exposure, may be helpful to determine whether different conditions of exposure may affect epigenetic phenomena and the directions of the epigenetic alterations in relation to the qualitative and quantitative characteristics of the exposures.

From an occupational health perspective, future prospective studies, focusing on the identification of epigenetic alterations as possible biomarkers of exposure or early health effects should be planned. Longitudinal studies in this regard may be helpful to assess causal relationships and to avoid misinterpretation of data due to intrapersonal variation over time. Understanding the peculiar epigenetic pathways underlining welding fume action and their possible functional significance may further have implications for the development of exposure control measures, which may include workplace collective or individual protections as well as changes in lifestyle habits, aimed to prevent adverse, irreversible, health effects [35]. Moreover, we cannot exclude that, in the next future, as the link between epigenetic changes and specific phenotypes become more substantial, these molecular patterns may be tested, and validated as possible early effect biomarkers in occupationally exposed subjects. Moreover, they may be employed to define conditions of hyper-susceptibility to specifically hazardous workplace exposures. It this latter regard, it seems important that ethical considerations relative to the possible adverse impact on the worker’s status of employment and/or quality of life, should be always taken into account while planning to use these biomarkers to assess the workers’ fitness to their jobs.

## 5. Conclusions

Current evidence does not allow to definitively extrapolate a causal relationship between welding fumes and early epigenetic marker changes. However, the positive results obtained with certain epigenetic alterations, following exposure to well-known hazardous welding fumes, and the public health relevance of possible welding fume associated disease manifestations, require further scientific efforts. The biological mechanisms underlining epigenetic phenomena, whether and how methylation may affect gene expression, as well as the influence on disease development and progression remain to be elucidated. Future investigations should be focused at understanding epigenetic effects under low-dose, long-term conditions of exposure currently experienced in occupational settings, and define their role in eventually long-latency welding fume effects. To deeply define a possible dose-response relationship quantitative and qualitative environmental monitoring information, as well as internal dose biological monitoring data should be acquired, in order to reach a better comprehension of the exposure features influencing epigenetic responses and health effects. Overall, understanding epigenetic changes in response to welding fume exposure and their relationship with adverse health effects may be important to achieve a suitable risk assessment process. This may finally provide guidance to adequate risk management procedures to protect the health of exposed workers.

## Figures and Tables

**Table 1:** Epigenetic changes induced by welding fume exposure.

Investigated Population (Number); Age; Job Tasks	Setting	Period of Investigation	Methods	Epigenetic Outcomes	Main Results	Exposure Levels	Reference
Boilermakers (66 M) Mean age (range): 41 (21–71 years) Job tasks: metal arc and gas metal arc welding using base mild and stainless-steel metals with iron electrodes	Quincy, MA, USA	Recruitment period: January 2010–June 2012.	DNA methylation levels in Alu and LINE-1 elements in human genome was measured in peripheral blood leukocytes under ambient pre- and post-shift short term exposures (5-h exposure). Pre and post-welding resting HRV was recorded.	DNA Methylation	✓ Significant positive association between PM_2.5_ exposure and LINE-1 methylation. ✓ N.s. association between PM_2.5_ exposure and Alu methylation. ✓ N.s. association between LINE-1 methylation and HRV in exposed welders. ✓ PM_2.5_ exposure was associated with decreased HRV.	PM_2.5_ levels from the background environment in the union hall with no direct welding fume exposure (mean ± SD): 0.11 ± 0.14 mg/m^3^. Union hall was a large, temperature-controlled classroom with ten workstations in a separate area where welding, cutting and grinding tasks were performed. Personal real time PM_2.5_ exposure continuously monitored during welding (average of 5 h): 0.43 ± 0.34 mg/m^3^.	Fan et al. [28]
Welders in a boiler-maker union (75 M) Mean age (range): 41.6 (21.6–71.2 years) Job tasks: to assemble and weld boilers	Quincy, MA, USA	Recruitment period: January 2003–June 2012.	Urine, blood and 12 min resting ECG recordings were collected pre- and post-6-h work-shift. DNA methylation levels of the entire genome was assessed pre- and post-shift exposure	DNA Methylation	✓ Significant negative association between the methylation levels of the CpG (cg26829071) located in the GPR133 gene on chromosome 12 and DC. ✓ Significant association between the methylation of CpG (cg12991522) located in the PPL gene on chromosome 16 and DC. ✓ Significant association between methylation levels of CpG (cg15273468) located on chromosome 10 and AC.	Major activities included welding, grinding and cutting tasks. No data on welding PM concentrations were provided.	Zhang et al. [35]
Welders in a boiler-maker union (48 M)	Quincy, MA, USA	Recruitment period: January 2007–June 2012	Blood mtDNA methylation levels in mtDNA promoter (D-loop) and genes for ATP synthesis (MT-TF and MT-RNR1) was assessed by bisulfite pyrosequencing. A continuing Holter monitoring ECG recordings at both prior and post worked has been collected.	MtDNA methylation	✓ The mean level of DNA methylation within the D-loop region was 2.36% and 2.22% in pre- and post-welding work. ✓ Mean DNA methylation level of the D-loop region was significantly lower in participants who were highly exposed to PM. ✓ Mean levels of mtDNA methylation within the MT-TF and MT-RNR1 regions were not significantly associated with PM_2.5_ exposure. ✓ The mtDNA methylation from the D-loop and MT-TF and MT-RNR1 regions was not directly associated with markers of HRV. ✓ The interactions between PM_2.5_ and mtDNA methylation were significantly and positively associated with the measured HRV markers.	Personal real time PM_2.5_ exposure after a welding day (5 h): 0.38 mg/m^3^; PM_2.5_ levels from the background environment: 0.15 mg/m^3^. For details on background environment, see Fan et al. [28]	Byun et al. [48]
Welders from 10 companies (101 M); Unexposed controls (127 M) Mean age (range): 41 (23–60 years) Median span working as welders: 7 years Job tasks: gas metal arc welding with mild steel	Sweden	-	DNA was isolated from peripheral venous blood. F2RL3 methylation levels were assessed in CpG sites (CpG_2 to CpG_5) through pyrosequencing assays.	DNA Methylation	✓ Welders had a significantly lower methylation of the CpG_5 site in F2RL3 compared to controls. ✓ Welders had a significantly higher methylation of the CpG_2 and CpG_4 sites in F2RL3 compared to controls. ✓ Significant inverse association between methylation of CpG_4 and working years.	Respirable dust samples were collected from the workers’ breathing zone. Mean respirable dust exposure for welders (1.2 mg/m^3^) and controls (0.2 mg/m^3^). Unexposed controls were blue collars organizing grocery goods or gardeners.	Hossain et al. [40]
See Hossain et al. [40]	Sweden	-	Peripheral blood mtDNA copy number was measured by quantitative PCR and methylation levels in mtDNA promoter (D-loop) and tRNA encoding gene MT-TF was assessed by bisulfite-pyrosequencing. Blood pressure has been collected once for each subject.	MtDNA methylation	✓ RmtDNAcn was significantly higher in the welders (median value of 1.13) than in the controls (median value of 1.00). ✓ D-loop and MT-TF methylation was significantly lower in exposed welders (median values of 13.4 and 3.4, respectively) compared to controls (median values of 15.6 and 4.5, respectively). ✓ Significant negative association between D-loop methylation and RmtDNAcn. Not significant correlation between MT-TF methylation and RmtDNAcn. ✓ Higher systolic blood pressure in in the welder group with low mtDNA function indicated by a lower number of copies of active unmethylated D-loop or MT-TF.	Mean personal respirable dust (50% cut-off at an aerodynamic equivalent particle diameter of 4 μm) exposure level: 1.1 mg/m^3^ for welders (6.8 h workday); 0.1 mg/m^3^ for controls. Engineered exposure control measures: local exhaust ventilation (55% of welders). PPE adopted: welding shields (56%); powered air purifying respirators (44%); protective clothing (100%).	Xu et al. [49]
Boilmakers (38 M) Mean age (range): 35.6 (21.3–61 years) Average years worked (range): 6.8 (1–35 years) Job tasks: cutting and welding metal plates through different welding technologies (oxyacetylene gas torches, gas tungsten arc, shielded metal arc, gas metal arc welding) on mild and stainless steel bases.	USA	Recruitment period: 2003, 2008	DNA methylation levels in Alu, and LINE 1 human genome elements, as well as in the promoter region of the iNOS gene was measured in peripheral blood cells under ambient pre- and post-shift short term exposures (6 h acute exposure). Whole blood samples were collected prior of any welding activities (pre-shift) and immediately after the exposure period (post-shift). Blood samples were collected from individual who welded during the day of sampling (82%) or who were only present in the practice hall (18%). DNA methylation was associated also to years worked as boilermakers (chronic exposure)	DNA Methylation	✓ Average percentages of methylated cytosines at baseline and post-shift for Alu, LINE-1 and iNOS: 25.5%, 85.3%, 97.5% and 25.5%, 85.4%, 97.3% respectively. ✓ Significant positive association between PM_2.5_ levels in a single work-shift of exposure, and in chronic exposures (assessed as the number of years worked as boilermakers) and increased methylation in the promoter region of the iNOS gene. ✓ No associations between PM_2.5_ exposure and Alu or LINE-1 methylation.	Welding was performed in a room outfitted with 10 workstations, each with local exhaust ventilation. Mean work shift PM_2.5_ exposure: 1.06 mg/m^3^ (average acute duration of exposure: 303.2 min). In 13 instances respirators were used by workers during sampling.	Kile et al. [34]
Welders (201 M) Mean age (range): 25–65 years Average years worked (mean ± standard deviation): 19.8 ± 13.8 years Job tasks: job with exposure to welding fumes/welders	USA	-	Workers were categorized as parkinsonism cases (n. 49); intermediate cases (n. 49); controls (n. 103). NOS2 DNA methylation was assessed at three CpG sites: 8309, 8314, 8329 located in exon 1 bordering the 5′ promoting region. DNA was isolated from peripheral venous blood.	DNA Methylation	✓ A significant inverse association between NOS2 methylation and Parkinsonism was evident. ✓ Compared to the lowest tertile of NOS2 methylation, the prevalence of parkinsonism was 59% lower in the middle tertile of NOS2 methylation and 76% lower in the upper tertile of NOS2 methylation. ✓ The association between parkinsonism and NOS2 methylation was most evident for the CpG site 8329. ✓ An inverse association between total duration of welding fume exposure and NOS2 8329 methylation among workers with <10 years of cumulative exposure (0.16% lower methylation per year); no association otherwise.	-	Searles Nielsen et al. [56]
See Hossain et al. 2015	Sweden	-	Venous blood and spot urine samples were collected from each participant. Blood was collected during the last 4 h of an 8-h work shift throughout the week. DNA was isolated from whole peripheral blood. Quantitative polymerase chain reaction (PCR) was used to determine relative telomere length. Methylation status of 10 tumor suppressor genes was determined by methylation-sensitive high-resolution melting analysis.	DNA Methylation	✓ Welders and controls did not differ in 8-oxodG-levels or relative telomere length in adjusted models. ✓ DNA methylation index (low, one gene; medium, two genes; high, three or more genes): 39/51/30 in controls; 26/34/28 in welders (n.s). ✓ Significantly higher methylation of the adenomatous polyposis coli (APC) gene was detected in welders compared to controls, although in a fully-adjusted model, such difference was not significant.	Respirable dust samples (50% cut-off at an aerodynamic equivalent particle diameter of 4 μm) were collected within the workers’ breathing zone. Average sampling time was 6.8 h (range: 2.4–8.6). Mean respirable dust exposure: 1.2 mg/m^3^ for welders; ≤0.1 mg/m^3^ for controls. PPE adopted: powered air purifying respirators (50% of welders).	Li et al. [52]

AC, acceleration capacity; DC, deceleration capacity; HRV, heart rate variability; n.s., not significant; PM, particulate matter; PPE, personal protective equipment.
